# Randomized trial examining effectiveness of lifestyle intervention in reducing gestational diabetes in high risk Chinese pregnant women in Hong Kong

**DOI:** 10.1038/s41598-018-32285-6

**Published:** 2018-09-14

**Authors:** Ruth Suk-Mei Chan, Wing-Hung Tam, Ivan Chak-Hang Ho, Macy Wai-Chi Kwan, Liz Sin Li, Mandy Man-Mei Sea, Jean Woo

**Affiliations:** 1 Department of Medicine and Therapeutics, The Chinese University of Hong Kong, Hong Kong, China; 2 Department of Obstetrics and Gynaecology, The Chinese University of Hong Kong, Hong Kong, China; 3 Centre for Nutritional Studies, The Chinese University of Hong Kong, Hong Kong, China

## Abstract

Gestational diabetes mellitus (GDM) is of public health concern. This trial examined whether a clinically proven lifestyle modification program (LMP) in early pregnancy was superior to routine antenatal care in improving GDM, maternal and infant outcomes. Chinese pregnant women at risk of GDM (n = 220) were recruited at or before 12-week gestation and randomized to either a LMP group or a routine care control group. Eighty subjects completed a dietitian-led LMP including dietary and exercise components from early pregnancy till 24-week gestation. Data were compared with those of 86 control subjects. Twenty three (26.7%) control subjects and 20 (25.0%) LMP subjects developed GDM (*p* = 0.798). The proportion of infants born large for gestational age and macrosomia was similar between groups. The LMP group showed a lower proportion of excessive gestational weight gain (GWG). Subgroup analysis suggested that those with higher LMP adherence showed more desirable dietary composition and energy intake, and lower proportion of excessive GWG compared with the low LMP adherence group and the control group. The potential effect of LMP on GDM and other maternal and infant outcomes, in particular GWG, as well as barriers for making lifestyle changes warrant further investigations (ClinicalTrials.gov NCT02368600).

## Introduction

Gestational diabetes mellitus (GDM) is defined as a type of diabetes first diagnosed during pregnancy^1^. With increasing prevalence of obesity and diabetes worldwide, the number of women with GDM is also increasing^2^. Women with GDM have an increased risk developing metabolic syndrome^3^ and vascular diseases^4^. GDM has also been linked with higher rates of cesarean sections, induced deliveries, shoulder dystocia and macrosomia, as well as predisposes the offspring to overweight and metabolic syndrome^4,5^. These observations highlight the importance of timely intervention in the prenatal period in reducing the lifetime burden from non-communicable diseases.

Currently there is no consensus regarding the best intervention for weight management and GDM reduction during pregnancy^6^. Available evidence appears to suggest that antenatal lifestyle interventions, especially dietary interventions are associated with restricted gestational weight gain (GWG) and could possibly reduce the risk of GDM in overweight or obese pregnant women^7^. However, most available trials that have been conducted in Western and Chinese populations focused on the treatment of GDM and only few of them have tested the effectiveness of lifestyle interventions using the prevention of GDM as the primary outcome^8,9^. Therefore, well designed randomized trials with standardized behavioural interventions are warranted.

This trial examined the potential use of a clinically proven lifestyle modification program (LMP)^10,11^ in early pregnancy in preventing GDM in Chinese pregnant women at high risk of GDM in Hong Kong. The proposed intervention was unique that it was offered at or before 12 weeks of gestation, and such design supported the importance of early intervention in pregnancy in reducing GDM and preventing excessive GWG. We aimed to compare the effectiveness of a lifestyle intervention in early pregnancy with usual antenatal care in reducing the GDM incidence (primary outcome), decreasing the proportion of infants born large for gestational age (LGA) and being classified as macrosomia (secondary outcomes), as well as improving other maternal and birth outcomes (tertiary outcomes) in Chinese pregnant women at risk of GDM in Hong Kong. We hypothesized that the proposed lifestyle invention was superior to usual antenatal care in improving all these outcomes.

## Materials and Methods

### Study design and study population

This was a prospective parallel group, single-blind randomized controlled trial (RCT), which was conducted between April 2015 and April 2017 (Supplement 1). Chinese women with age of 18 years old and above and having a gestational age <=12 weeks were recruited using a convenience sampling method at the antenatal clinic of a study hospital in Hong Kong. Research staff screened clients attending the clinic and identified eligible participants.

Women should fulfil at least one of the hospital criteria of defining as at risk of GDM upon recruitment. The criteria included maternal age >=35 years old at the expected date of confinement; prior history of GDM or birth of child >=4 kg; pre-pregnant body mass index (BMI) or BMI at the 1^st^ trimester >=25 kg/m^2^; and family history of diabetes at the first degree relatives. Those who were participating in any clinical trial, had pre-existing diabetes, multiple pregnancies, substance abuse, renal, liver or thyroid dysfunction, cognitive impairment, or any other indication of major medical or psychological illnesses, as well as physical restriction that led to exercise avoidance were excluded. All participants provided written informed consent. The study protocol was performed in compliance with the Declaration of Helsinki and was approved by the Clinical Research Ethics Committee of The Chinese University of Hong Kong. The study was registered at ClinicalTrials.gov (NCT02368600, registered date: 15/02/2015).

Eligible participants were randomized in 1:1 ratio to either the intervention group or the control group upon recruitment. Randomization was performed through the use of a computer-generated list of random numbers in blocks of 6 by a study coordinator. Treatment assignments were concealed in consecutively-numbered sealed envelopes, which were opened sequentially upon subject enrollment. The interventionists (the dietitian and the exercise instructor), the participants and the study coordinator were not blinded to the treatment assignment. However, the interventionists did not take any outcome measurements. All investigators, outcome assessors, clinicians and nurses of routine antenatal and postnatal care were blinded to the treatment assignment.

### Intervention group (LMP group)

On top of routine antenatal care, participants in the intervention group participated in a dietitian-led lifestyle intervention from the first antenatal booking (i.e. <=12 weeks of gestation) to 24 weeks of gestation. The intervention was designed based on a clinically proven LMP^11^. Participants received bi-weekly face-to-face or phone consultations in the first 2 months and monthly face-to-face consultations afterwards till the end of the intervention (i.e. 24–28 weeks of gestation). At the first 1-hour face-to-face session, the dietitian comprehensively reviewed the participant’s lifestyle habits, medical, pregnant and birth history, as well as the fetal growth status and the participant’s weight gain of current pregnancy, and discussed the specific dietary and lifestyle advices to achieve a desirable weight status with the participant. In the follow-up consultations through face-face interviews or phone calls (~20 minutes), the dietitian reviewed the participant’s dietary and lifestyle practices and provided recommendations. Each participant was given an individualized menu plan and healthy lifestyle booklets aiming at achieving a varied balanced diet with an emphasis on fruit and vegetables consumption, and intake of moderate-carbohydrate, low-fat, low-glycemic index (GI) and low-calorific products in appropriate portions. The diet plan was also designed to achieve a desirable fetal growth and maternal weight throughout pregnancy. Advice on the use of dietary supplements and managing pregnancy discomforts was given. Participants could email their enquiries to the dietitian.

Besides, participants were encouraged to see the exercise instructor at least once during the LMP. During the exercise consultation (~30 minutes), the exercise instructor reviewed participant’s medical history as well as pre-pregnant and current exercise habits, assessed participant’s fitness level and musculoskeletal problems, and designed a suitable exercise regime for the participant based on international guidelines^12,13^. Participants were generally advised to do a 30-minute of easy to moderate intensity of low impact aerobic exercise at least three times a week.

### Control group

The control group received routine antenatal care. In brief, body weight of pregnant woman attending the antenatal clinic was monitored at each antenatal visit by nurses. Educational booklet on diet and exercise recommendations during pregnancy was delivered to them. They were also offered optional antenatal classes which were subjected to the class availability.

### Study outcomes and sample size calculation

The primary outcome was the proportion of participants in each group with GDM at 24–28 weeks of gestation. The secondary outcomes included the proportion of neonates born with LGA (>90^th^ percentile of the customized birth weight) and macrosomia (>=4 kg at birth).

Sample size was calculated based on the primary outcome using data from a previous trial^14^. A sample size of 73 per group was calculated with power of 90% at a 1% alpha level and two-sided test to detect an 83% reduction in the odds of decreased GDM in the intervention group when compared with the control group^14^. Assuming 30% lost to follow-up rate and 5% miscarriage rate, a final sample size of 110 participants per group was decided.

### Measurements

Maternal and fetal/neonatal data were collected by trained research staff at different stages of pregnancy, after delivery and 6–8 weeks postpartum. A cash of HK$50 (i.e. about 6 US dollars) was given to the participants for the completion of assessments at designated time points.

Data on demographics, lifestyle habits, as well as medical and obstetric history were collected using a standardized questionnaire at baseline (i.e. <=12 weeks of gestation). Maternal weight, height and blood pressure were measured using standardized methods at various time points, and pre-pregnant weight was self-reported. The total GWG was calculated as the difference in the weight measured on the day of delivery and the self-reported pre-pregnant weight.

A 3-day diet record was used to assess subject’s diet at baseline and 24–28 weeks of gestation. Daily nutrient intake and consumption of food group of each subject were calculated using the nutrition analysis software Food Processor Nutrition analysis and Fitness software version 8.0 (ESHA Research, Salem, USA) which included nutrient data of local foods from food composition tables from China and Hong Kong. Since no validated Chinese questionnaire for measuring physical activity level for pregnant women was available, the Chinese version of the International Physical Activity Questionnaire (IPAQ-C)^15^ was used to assess the physical activity level. A diet adherence score and a physical activity adherence score were generated based on the 3-day diet record and the IPAQ-C respectively for the participants in the LMP group at baseline and 24–28 weeks of gestation. The diet adherence score consisted of eight criteria and one score was given for meeting each of the criteria: (i) total energy not exceeding 10% of the diet plan; (ii)% energy from fat 20–30%; (iii)% energy from protein within the range of 15–20%; (iv)% energy from carbohydrate 50–60%; (v) consumption of fruit ≥160 g; (vi) consumption of vegetables ≥240 g; (vii) regular meal consumption and (viii) “Avoid food (e.g. high fat or high sugar foods/GI foods)” not being consumed. The total score ranged from 0 to 24 for each time point. Adherence to the LMP was also assessed based on the percentage attendance to the proposed LMP sessions. The physical activity adherence score was assessed based on the IPAQ-C data. Two scores were given if 80% of the recommended volume of exercise (frequency x duration) was met during the week of follow-up (7 days) while one score was given if 50% of the recommended volume of exercise was achieved. Zero score was given for those who did not perform any easy to moderate intensity of low impact aerobic exercise. Number of exercise consultation sessions attended by the participants was also documented.

Participants in the intervention group were asked to rate the perceived support from the dietitian and the exercise instructor at 24–28 weeks of gestation using the validated Chinese version of Health Care Climate Questionnaire (HCCQ)^16^. HCCA is a 15-item patient-rated measure related to the perceived supportiveness of health care providers. Each item has a scale of 1 (strongly disagree) to 7 (strongly agree). A total rated score can be generated with a range of 1 (lowest perceived support) to 7 (highest perceived support). A higher rated total score indicates higher perceived support.

A 75 g 2-h oral glucose tolerance test (OGTT) was done at 24–28 weeks of gestation on routine care basis. GDM was diagnosed using the modified World Health Organization criteria in which one or more of the criteria were met: (i) fasting plasma glucose 5.1–6.9 mmol/L; (ii) 1-hour plasma glucose >=10 mmol/L; or (iii) 2-hour plasma glucose 8.5–11.0 mmol/L following a 75 g oral glucose load^17^. OGTT was repeated at 6–8 weeks postpartum for those who had GDM at 24–28 weeks of gestation.

Perinatal and obstetric outcomes, neonatal outcomes and complications were retrieved from the hospital record. Perinatal and obstetric outcomes and complications included preeclampsia, gestational hypertension, Caesarean section and preterm delivery, and gestation at delivery (week). Fetal and neonatal outcomes included Apgar score at 5 minutes, neonatal weight, small for gestational age (SGA), LGA, macrosomia, and shoulder dystocia.

### Data analysis

Data were presented as mean (standard deviation, SD) for normally distributed variables, median (interquartile range, IQR) for skewed variables and frequency (percentage) for categorical variables where appropriate. Between-groups comparisons at baseline and after intervention were made using the Student’s t test, Chi-square test or Fisher’s exact test where appropriate. Data were analysed using both the intention-to-treat (ITT) approach and the per-protocol (PP) approach. The compliance to the LMP protocol were assessed based on the adherence scores and the attendance data. Those who achieved at least 50% of the diet adherence score as well as attending at least 70% of the dietetic consultations and at least one exercise consultation were defined as high LMP adherent cases for the PP analysis. The physical activity adherence score was finally excluded from the criteria for PP analysis because majority of the LMP participants had zero physical activity adherence score. Regression techniques were used to examine the influence of prognostic factors on the major outcomes. Multivariate regression models including logistic regression, linear regression, analysis of covariance, and multinomial regression were used to compare differences between the 2 groups for all measured outcomes at baseline and 24–28 weeks of gestation or at delivery with adjustment for potential covariates. Potential covariates included maternal age (continuous), marital status (married vs. others), family monthly income (less than HK$ 20,000 vs. HK$ 20,000 or above), and pre-pregnant BMI (continuous). They were mainly chosen based on *p* < 0.2 in the univariate analysis. All statistical tests were two-sided and a p-value < 0.05 was considered statistically significant. SPSS for Windows software (version 24.0, SPSS Inc., Chicago, IL, USA) was used for the statistical analysis.

## Results

### Characteristics of the participants

**Figure 1:**
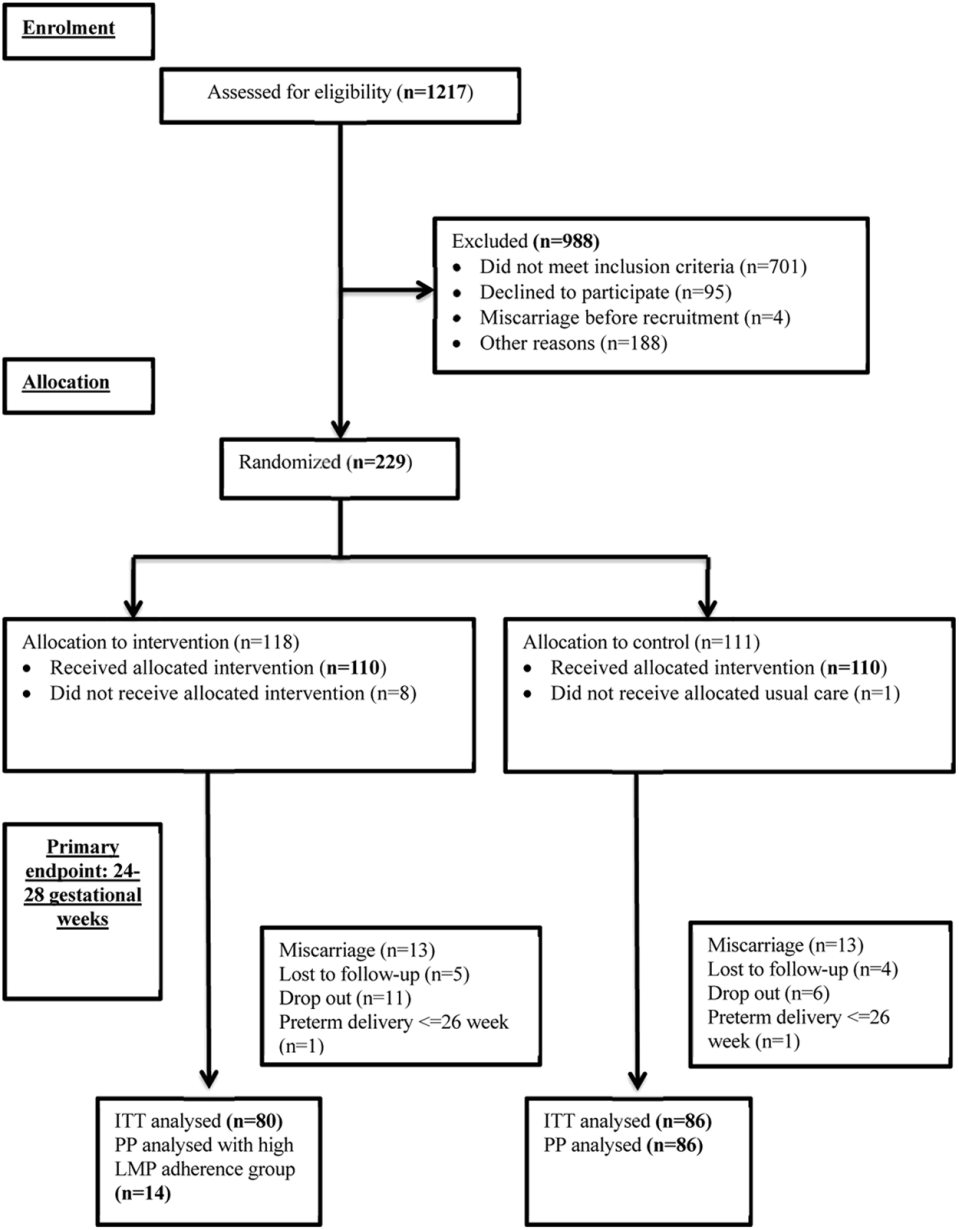
ITT - intention to treat. PP – per protocol. LMP – lifestyle modification program.

Figure 1 shows the number of participants at different study stages. Participants who were classified as loss to follow-up or drop out (n = 26) were more likely to be ex-smokers (n = 5, 19.2%) in comparison to those who remained in the study (n = 11 out of 194, 5.7%) (*p* = 0.027). The mean (SD) age and BMI of the final 166 participants were 33.1 (4.2) years and 23.6 (4.0) kg/m^2^ respectively. The gestational age at enrolment ranged from 4 to 11 weeks and 59.6% of them were nulliparous. Baseline characteristics were comparable between the two study groups (Table 1).

**Table 1:** Baseline characteristics of study participants (n = 166).

Characteristics at enrolment	LMP group (n = 80)	n(%)	Control group (n = 86)	n(%)	P value^†^
	Mean ± SD		Mean ± SD		
Age (years)	33.2 ± 4.4		33.1 ± 4.1		0.801
Gestational week	6.8 ± 1.6		6.8 ± 1.5		0.942
Body weight (kg)	57.8 ± 10.8		59.9 ± 9.9		0.200
Body height (cm)	158.2 ± 5.5		157.8 ± 5.1		0.674
BMI (kg/m^2^)	23.1 ± 3.9		24.1 ± 4.0		0.104
Pre-pregnant weight (kg)	56.6 ± 10.5		58.1 ± 9.6		0.327
Pre-pregnant BMI (kg/m^2^)	22.6 ± 3.8		23.4 ± 3.9		0.191
Family history of diabetes at 1^st^ degree relatives		29(36.3%)		28(32.6%)	0.617
Prior history of GDM or birth of child >=4 kg		1(1.3%)		0(0.0%)	0.482
**Education level**					
Secondary or below		31(38.8%)		41(47.7%)	0.246
Tertiary or above		49(61.2%)		45(52.3%)	
**Occupation status**					
Working		70(87.5%)		71(82.6%)	0.374
Non-working^‡^		10(12.5%)		15(17.4%)	
*Marital status*					
Married		70(87.5%)		81(94.2%)	0.133
Others^§^		10(12.5%)		5(5.8%)	
**Monthly family income (HK$)**					
Less than 20,000		13(16.2%)		26(30.2%)	**0**.**034**
20,000 or above		67(83.8%)		60(69.8%)	
**Parity**					
0		49(61.2%)		50(58.1%)	0.683
1 or more		31(38.8%)		36(41.9%)	
**Smoking** ^¶^					
Never smoke		76(95.0%)		83(96.5%)	0.712
Ex-smoker		4(5.0%)		3(3.5%)	
**Alcohol use**					
Never		65(81.2%)		74(86.0%)	0.403
Ex-user/current user^¶¶^		15(18.8%)		12(14.0%)	

LMP – lifestyle modification program. SD – standard deviation. BMI – body mass index. GDM – gestational diabetes mellitus. ^†^P value by independent t test or Chi square/Fisher’s exact test where appropriate. ^‡^Non-working included unemployment and housewife. ^§^Others included never married, widowed, separated, and divorced. ^¶^No current smoker in both the LMP group and the control group. ^¶¶^Only one subject in the LMP group was a current alcohol user.

### Compliance of the LMP group

Overall, 93.8% participants attended at least 70% of the dietetic consultations and 92.5% participants attended at least one exercise consultation. Diet adherence score ranged from 2 to 18, with mean (SD) of 9 (3). Physical activity adherence score ranged from 0 to 2, with median (IQR) of 0 (0-0). Mean (SD) scores of perceived support from the dietitian and the exercise instructor were 5.8 (0.6) and 5.4 (0.7) respectively. Fourteen participants in the LMP group were classified as high LMP adherence group and they were included for PP analysis.

Dietary intakes were similar between the two groups (Table 2). The magnitude of increase in energy intake was however significantly smaller in the LMP group than the control group. The high LMP adherence group also showed a significantly smaller magnitude of increase in energy intake than the control group in the PP analysis. The high LMP adherence group showed a trend of adopting a more balanced diet in terms of the energy contribution from various macronutrients (i.e. reduced percentage energy from total fat and saturated fat) after the intervention compared with the control group (Table 3). In subgroup analysis, the high LMP adherence group shifted to a more balanced diet (i.e. reduced percentage energy from total fat and saturated fat) compared with the low LMP adherence group, although the magnitude of increase in energy intake was similar between the two groups (details not shown).

**Table 2:** Comparisons of energy and selected nutrient intakes as well as physical activity levels between the LMP group and the control group at <=12 weeks (T0) and 24–28 weeks (T2) (ITT analysis)^†^.

Variable	Weeks of gestation	LMP group (n = 80)	Control group (n = 86)	*P* value^‡^
Energy (kcal/day)	<=12 weeks	1775 ± 342	1652 ± 364	0.051
	24–28 weeks	1865 ± 386	2015 ± 434	**0**.**040**
	Difference^§^	90 ± 417	363 ± 428	**<0**.**001**
% of carbohydrates	<=12 weeks	45.0 ± 6.5	43.7 ± 6.3	0.248
	24–28 weeks	43.7 ± 5.5	43.1 ± 6.9	0.596
	Difference	−1.2 ± 7.0	−0.5 ± 8.0	0.592
% of fat	<=12 weeks	36.9 ± 5.3	37.3 ± 5.0	0.679
	24–28 weeks	37.2 ± 4.1	37.9 + 5.7	0.385
	Difference	0.3 ± 5.8	0.7 ± 7.1	0.747
% of saturated fat	<=12 weeks	9.4 ± 1.9	9.3 ± 1.6	0.740
	24–28 weeks	9.4 ± 1.6	9.9 ± 1.9	0.113
	Difference	0.0 ± 2.1	0.6 ± 2.3	0.119
% of protein	<=12 weeks	18.3 ± 2.4	19.3 ± 2.7	**0**.**036**
	24–28 weeks	19.4 ± 2.6	19.1 ± 3.6	0.623
	Difference	1.1 ± 2.8	−0.1 ± 3.7	**0**.**036**
Fiber (g/1000 kcal)	<=12 weeks	6.4 ± 2.2	6.2 ± 1.9	0.631
	24–28 weeks	6.7* + *1.7	6.4 ± 2.0	0.350
	Difference	0.3 ± 2.3	0.2 ± 1.8	0.719
Total PA (MET-minutes/week)	<=12 weeks	693(330–2079)	990(495–2153)	0.155
	24–28 weeks	693(476–1626)	1386(644–2772)	**0**.**030**
	Difference	16.5(−693–417)	165(−378–1047)	0.105
Vigorous (MET-minutes/week)	<=12 weeks	0(0–0)	0(0–0)	0.975
	24–28 weeks	0(0–0)	0(0–0)	0.086
	Difference	0(0–0)	0(0–0)	0.180
Moderate (MET-minutes/week)	<=12 weeks	0(0–0)	0(0–0)	0.054
	24–28 weeks	0(0–0)	0(0–0)	0.211
	Difference	0(0–0)	0(0–0)	0.917
Walking (MET-minutes/week)	<=12 weeks	693(297–1617)	924(495–2030)	0.198
	24–28 weeks	693(446–1386)	1188(495–2772)	0.125
	Difference	99(−693–446)	0(−421–767)	0.467
Sitting time (minutes/day)	<=12 weeks	447 ± 182	448 ± 218	0.976
	24–28 weeks	387 ± 174	404 ± 208	0.606
	Difference	−60 ± 140	−44 ± 209	0.601

Data are expressed as mean ± SD or median (IQR). ITT – intention to treat. LMP – lifestyle modification program. SD – standard deviation. IQR – interquartile range. PA – physical activity. MET – metabolic equivalent of tasks. ^†^Dietary data are presented based on 61 LMP participants and 69 control participants with complete dietary data at both <=12 weeks and 24–28 weeks. Physical activity data are presented based on 67 LMP participants and 69 control participants with complete physical activity data at both <=12 weeks and 24–28 weeks. ^‡^P value by independent t test or non-parametric Mann-Whitney test where appropriate. ^§^Difference based on T2 (24–28 weeks) value minus T0 (<=12 weeks) value.

**Table 3:** Comparisons of energy and selected nutrient intakes as well as physical activity levels between the high LMP adherence group and the control group at <=12 weeks (T0) and 24–28 weeks (T2) (PP analysis)^†^.

Variable	Weeks of gestation	LMP group (n = 14)	Control group (n = 86)	*P* value^‡^
Energy (kcal/day)	<=12 weeks	1704 ± 317	1652 ± 364	0.620
	24–28 weeks	1814 ± 319	2015 ± 434	0.104
	Difference^§^	110 ± 395	363 ± 428	**0**.**045**
% of carbohydrates	<=12 weeks	44.8 ± 9.3	43.7 ± 6.3	0.577
	24–28 weeks	46.9 ± 6.2	43.1 ± 6.9	0.068
	Difference	2.1 ± 9.3	−0.5 ± 8.0	0.287
% of fat	<=12 weeks	36.3 ± 7.5	37.3 ± 5.0	0.548
	24–28 weeks	34.8 ± 4.9	37.9 ± 5.7	0.060
	Difference	−1.5 ± 7.7	0.7 ± 7.1	0.317
% of saturated fat	<=12 weeks	8.7 ± 2.1	9.3 ± 1.6	0.240
	24–28 weeks	8.7 ± 1.6	9.9 ± 1.9	**0**.**027**
	Difference	0.0 ± 2.3	0.6 ± 2.3	0.360
% of protein	<=12 weeks	19.0 ± 2.7	19.3 ± 2.7	0.743
	24–28 weeks	18.7 ± 2.3	19.1 ± 3.6	0.700
	Difference	−0.3 ± 2.6	−0.1 ± 3.7	0.904
Fiber (g/1000 kcal)	<=12 weeks	6.7 ± 2.1	6.2 ± 1.9	0.382
	24–28 weeks	7.2 ± 2.2	6.4 ± 2.0	0.191
	Difference	0.5 ± 3.1	0.2 ± 1.8	0.751
Total PA (MET-minutes/week)	<=12 weeks	594(309–1386)	990(495–2153)	0.175
	24–28 weeks	693(169–1782)	1386(644–2772)	0.079
	Difference	8(−703–276)	165(−378–1047)	0.292
Vigorous (MET-minutes/week)	<=12 weeks	0(0–0)	0(0–0)	0.652
	24–28 weeks	0(0–0)	0(0–0)	0.430
	Difference	0(0–0)	0(0–0)	0.646
Moderate (MET-minutes/week)	<=12 weeks	0(0–0)	0(0–0)	0.168
	24–28 weeks	0(0–0)	0(0–0)	0.176
	Difference	0(0–0)	0(0–0)	0.772
Walking (MET-minutes/week)	<=12 weeks	594(309–1386)	924(495–2030)	0.252
	24–28 weeks	693(169–1782)	1188(495–2772)	0.175
	Difference	8(−703–276)	0(−421–767)	0.563
Sitting time (minutes/day)	<=12 weeks	414 ± 179	448 ± 218	0.578
	24–28 weeks	347 ± 165	404 ± 208	0.341
	Difference	−66 ± 117	−44 ± 209	0.705

Data are expressed as mean ± SD or median (IQR). PP – per protocol. LMP – lifestyle modification program. SD – standard deviation. IQR – interquartile range. PA – physical activity. MET – metabolic equivalent of tasks.^†^Dietary and physical activity data are presented based on 14 LMP participants and 69 control participants with complete dietary and physical activity data at both <=12 weeks and 24–28 weeks. ^‡^P value by independent t test or non-parametric Mann-Whitney test where appropriate. ^§^Difference based on T2 (24–28 weeks) value minus T0 (<=12 weeks) value.

Baseline physical activity level was similar between the LMP group and the control group. Walking accounted for the majority of daily physical activities in both groups. There was no significant between-group difference in the change of physical activity variables (Table 2). Similar results were observed for the PP analysis (Table 3). Subgroup analysis showed no significant difference in the physical activity variables between the high LMP adherence group and the low LMP adherence group (details not shown).

### Study outcomes

There was no significant difference in the GDM incidence and the total GWG between the LMP group and the control group. No significant difference in LGA, macrosomia and other maternal and infant outcomes was observed between the two groups (Table 4). However, pre-pregnant BMI was found to be a significant factor for both the GDM incidence and the total GWG in the adjusted models (details not shown). Higher pre-pregnant BMI was associated with higher risk of GDM incidence [OR (95% CI): 1.12 (1.02 to 1.23), *p* = 0.022] and lower total GWG [Beta (95% CI): −0.426 (−0.631 to −0.220), *p* < 0.001].

**Table 4:** Study outcomes between the LMP group and the control group (ITT analysis).

Outcomes	LMP group		Valid n^†^	Control group		Valid n	LMP vs. control		Crude P value	LMP vs. control		Adjusted P value
	n(%)	Mean ± SD		n(%)	Mean ± SD		Crude OR/Mean difference^‡^	95% CI		Adjusted OR/Mean difference^§^	95% CI	
**Primary outcome**												
GDM at 24–28 weeks	20(25.0%)	—	80	23(26.7%)	—	86	0.91	0.46–1.83	0.798	1.00	0.48–2.08	0.993
**Secondary outcomes**												
SGA (<10^th^ percentile)	12(15.6%)	—	77	12(14.3%)	—	84	1.11	0.47–2.64	0.817	1.21	0.48–3.01	0.688
LGA (>90^th^ percentile)	8(10.4%)	—	77	6(7.1%)	—	84	1.51	0.50–4.56	0.468	1.53	0.48–4.87	0.469
Macrosomia (birth weight >=4 kg)	1(1.3%)	—	78	2(2.4%)	—	85	0.54	0.05–6.06	0.617	0.66	0.05–8.27	0.745
**Other maternal or delivery outcomes**												
Preeclampsia	0(0.0%)	—	77	1(1.2%)	—	84	—^¶¶¶^	—	—	—	—	—
Gestational hypertension	2(2.6%)	—	77	1(1.2%)	—	84	2.21	0.20–24.91	0.520	2.59	0.19–35.24	0.475
Caesarean section^¶^	24(34.8%)	—	69	16(20.5%)	—	78	2.07	0.99–4.33	0.054	2.20	1.00–4.84	**0**.**049**
Preterm delivery	3(3.8%)	—	78	0(0.0%)	—	84	—^¶¶¶^	—	—	—	—	—
Total gestational weight gain (kg)	—	11.6 ± 4.0	76	—	11.8 ± 5.9	81	−0.25	−1.85–1.35	0.758	−0.72	−2.28–0.85	0.368
Excessive gestational weight gain^¶¶^	14(18.4%)	—	76	21(25.9%)	—	81	0.65	0.30–1.39	0.261	0.65	0.28–1.47	0.297
Total gestational weight gain categories^¶¶^									0.491			0.537
Below recommended range	32(42.1%)	—	76	33(40.7%)	—	81	1			1		
Within recommended range	30(39.5%)	—		27(33.3%)	—		1.15	0.56–2.34		1.13	0.53–2.40	
Above recommended range	14(18.4%)	—		21(25.9%)	—		0.69	0.30–1.58		0.67	0.28–1.64	
**Other infant outcomes**												
Gestation age at birth (week)	—	39.1 ± 1.2	78	—	39.2 ± 1.0	84	−0.03	−0.37–0.31	0.849	−0.02	−0.37–0.33	0.907
Birth weight (g)	—	3131 ± 441	78	—	3128 ± 361	85	3.74	−120.55–128.03	0.953	32.08	−95.38–159.53	0.620
Birth weight <2500 g	3(3.8%)	—	78	2(2.4%)	—	85	1.66	0.27–10.21	0.584	1.27	0.18–8.80	0.809
Apgar score at 5 min <7	0(0.0%)	—	77	1(1.2%)	—	82	—^¶¶¶^	—	—	—	—	—
Shoulder dystocia	0(0.0%)	—	77	0(0.0%)	—	83	—^¶¶¶^	—	—	—	—	—

ITT – intention to treat. LMP – lifestyle modification program. SD – standard deviation. OR – odds ratio. CI – confidence interval. GDM – gestational diabetes mellitus. SGA – small for gestational age. LGA – large for gestational age. ^†^Valid n for the particular variable and the % of the categorical variable was calculated based on the valid n. ^‡^Control as the reference category. ^§^Adjusted for age (continuous), marital status (married vs. others), monthly family income (<HK$20,000 vs. >=HK$20,000) and pre-pregnant BMI (continuous). ^¶^Excluding those caesarean sections in private hospital due to maternal request (n = 16). ^¶¶^Based on US Institute of Medicine^28^. ^¶¶¶^Not applicable due to zero number of outcome variables.

Among the 43 subjects who developed GDM at 24–28 weeks of gestation, thirty-nine (19 LMP, 20 control) repeated OGTT at 6–8 weeks postpartum. There were 9 (45%), 4 (20%) and 7 (35%) subjects in the control group with normal glucose, impaired fasting glucose and impaired glucose tolerance status respectively whereas the number (%) was 8 (42.1%), 5 (26.3%) and 6 (31.6%) respectively in the LMP group (*p* = 0.895).

Although there were no significant differences in the rates of GDM and most outcomes between the high LMP adherence group and the control group, the former showed a significantly better control in the total GWG than the latter in both the crude and adjusted models (Table 5). Similarly, most study outcomes were not affected by the LMP adherence. However, the high LMP adherence group tended to show a lower proportion of excessive GWG compared with the low LMP adherence group (0% vs. 21.3%, *p* = 0.099) (details not shown).

**Table 5:** Study outcomes between the high LMP adherence group and the control group (PP analysis).

Outcomes	LMP group		Valid n^†^	Control group		Valid n	LMP vs. control		Crude P value	LMP vs. control		Adjusted P value
	n(%)	Mean ± SD		n(%)	Mean ± SD		Crude OR/Mean difference^‡^	95% CI		Adjusted OR/Mean difference^§^	95% CI	
**Primary outcome**												
GDM at 24–28 weeks	1(7.1%)	—	14	23(26.7%)	—	86	0.21	0.03–1.70	0.144	0.30	0.04–2.57	0.271
**Secondary outcomes**												
SGA (<10^th^ percentile)	1(7.1%)	—	14	12(14.3%)	—	84	0.46	0.06–3.86	0.476	0.53	0.06–4.76	0.573
LGA (>90^th^ percentile)	1(7.1%)	—	14	6(7.1%)	—	84	1.00	0.11–9.00	1.000	1.00	0.10–9.71	0.999
Macrosomia (birth weight >=4 kg)	0(0.0%)	—	14	2(2.4%)	—	85	—^¶¶¶^	—	—	—	—	—
**Other maternal or delivery outcomes**												
Preeclampsia	0(0.0%)	—	14	1(1.2%)	—	84	—^¶¶¶^	—	—	—	—	—
Gestational hypertension	0(0.0%)	—	14	1(1.2%)	—	84	—^¶¶¶^	—	—	—	—	—
Caesarean section^¶^	0(0.0%)	—	11	16(20.5%)	—	78	—^¶¶¶^	—	—	—	—	—
Preterm delivery	0(0.0%)	—	14	0(0.0%)	—	84	—^¶¶¶^	—	—	—	—	—
Total gestational weight gain (kg)	—	11.4 ± 3.0	13	—	11.8 ± 5.9	81	−0.37	−3.70–2.96	0.826	−1.34	−4.65–1.96	0.422
Excessive gestational weight gain^¶¶^	0(0.0%)	—	13	21(25.9%)	—	81	—^¶¶¶^	—	—	—	—	—
Total gestational weight gain categories^¶¶^									**0**.**016**			**0**.**049**
Below recommended range	5(38.5%)	—	13	33(40.7%)	—	81	1			1		
Within recommended range	8(61.5%)	—		27(33.3%)	—		1.96	0.57–6.68		1.59	0.43–5.90	
Above recommended range	0(0.0%)	—		21(25.9%)	—		0.00	0.00–0.00		0.00	0.00–0.00	
**Other infant outcomes**												
Gestation age at birth (week)	—	39.2 ± 0.8	14	—	39.2 ± 1.0	84	0.02	−0.56–0.59	0.953	0.03	−0.58–0.63	0.933
Birth weight (g)	—	3122 ± 291	14	—	3128 ± 361	85	−5.65	−207.4–196.1	0.956	1.32	−210.8–213.4	0.990
Birth weight <2500 g	0(0.0%)	—	14	2(2.4%)	—	85	—^¶¶¶^	—	—	—	—	—
Apgar score at 5 min <7	0(0.0%)	—	14	1(1.2%)	—	82	—^¶¶¶^	—	—	—	—	—
Shoulder dystocia	0(0.0%)	—	14	0(0.0%)	—	83	—^¶¶¶^	—	—	—	—	—

PP – per protocol. LMP – lifestyle modification program. SD – standard deviation. OR – odds ratio. CI – confidence interval. GDM – gestational diabetes mellitus. SGA – small for gestational age. LGA – large for gestational age. ^†^Valid n for the particular variable and the % of the categorical variable was calculated based on the valid n. ^‡^Control as the reference category. ^§^Adjusted for age (continuous), marital status (married vs. others), monthly family income (<HK$20,000 vs. >=HK$20,000) and pre-pregnant BMI (continuous). ^¶^Excluding those caesarean sections in private hospital due to maternal request (n = 10). ^¶¶^Based on US Institute of Medicine^28^. ^¶¶¶^Not applicable due to zero number of outcome variables.

## Discussion

Our study is one of the few available studies among Chinese pregnant women to examine the effectiveness of a lifestyle intervention in early pregnancy on GDM prevention. Unexpectedly, we failed to show any significant difference in any measured outcomes between the LMP group and the control group. Only a non-significant trend in limiting excessive GWG was observed in the LMP group. Our results were different from those reported by Sun and colleagues^9^ but consistent with the findings from other groups^18–21^ Sun *et al*. examined the effectiveness of a lifestyle intervention in early pregnancy on GDM prevention in 74 Chinese overweight and obese women (i.e. BMI >=24 kg/m^2^)^9^. The intervention included counselling sessions on diet, exercise and pregnant weight gain delivered by the research nurse at recruitment and on monthly basis in the second trimester plus weekly follow-up phone calls or emails between antenatal visits. The control group only received the same counselling at recruitment delivered by the research nurse and routine health education in the clinic. Lower incidence of GDM and lower excessive weight gain at the end of the second trimester were observed in the intervention group in comparison to the control group^9^. In contrast, other studies showed that lifestyle interventions did not alter the risk of GDM. A cluster RCT in Finland showed that individual intensive counselling on diet, physical activity and weigh gain offered by a nurse to pregnant women with at least one GDM risk factor within 8–12 weeks of gestation was effective in controlling neonatal birthweight and LGA but failed to reduce maternal GDM or limit GWG compared with the usual care control group. However, those who highly adhered to the intervention showed decreased risk of GDM and lower proportion of infants born with LGA compared with the usual care control group. About 60% of the overall sample in this Finnish study was overweight (i.e. BMI >25 kg/m^2^) at baseline^18^. In another RCT examining the effect of a lifestyle intervention including two phone dietary consultations and twice-weekly exercise groups on glucose metabolism in 606 healthy first time pregnant women with pre-pregnant BMI > = 19 kg/m^2^, the intervention failed to improve glucose levels or reduce GDM incidence but was able to reduce GWG at term as well as the insulin and leptin levels^20^. Similar lack of effect of lifestyle interventions on GDM prevention have been reported in other studies. However, the effectiveness of lifestyle interventions on other outcomes, such as limiting GWG or reducing proportion of infants born with LGA remains uncertain^19,21^.

Of particular interest is that those with higher adherence to the LMP in the present study showed a more desirable dietary energy intake and composition, and a lower proportion of excessive total GWG as compared with the control group and the low LMP adherence group. Meanwhile, low LMP adherence warrants concern. Although LMP participants reported a moderate to high perceived support from both dietitian and exercise instructor, previous studies suggested that some personal, social and environmental factors, such as social support, working environment and availability of healthy food choices may influence one’s food choices and physical activity behaviour during the lifestyle intervention^11,22^. Our observations possibly suggest that the LMP is potentially beneficial for bringing dietary changes only in those who have high motivation for lifestyle changes. Whether such lifestyle changes could lead to an ultimate reduction in the GDM incidence may require a trial with a larger sample size. Further studies are needed to identify barriers and best strategies to facilitate lifestyle changes in this high risk group.

Several reasons possibly explain the absence of effect of the LMP on the expected outcomes in our study. First, we recruited pregnant women at risk of GDM based on the hospital criteria and the average BMI at entry was about 23 kg/m^2^. Approximately 40% of them were overweight or obese at the time of enrolment. In contrast, other studies which were able to demonstrate the effectiveness of lifestyle interventions in reducing GDM incidence, limiting GWG or improving other maternal and neonatal outcomes mainly targeted overweight or obese women with pre-pregnant BMI of at least 24 kg/m^2^. Whether the LMP could produce a more pronounced effect on the outcomes in overweight or obese pregnant women remains to be explored. Second, the observed difference in GDM incidence between the LMP group and the control group for sample size calculation was smaller than expected. In contrast, the observed 12% miscarriage rate in the study was higher than that (i.e. 5%) used in the sample size calculation, thus the study may be underpowered. Third, the physical activity data showed that the exercise component of the LMP may need to be strengthened. Previous studies showed that supervised exercise programs might reduce GDM incidence^23,24^, limit GWG^23^, as well as improve some maternal and neonatal outcomes^24,25^. However, in traditional Chinese culture, pregnancy is considered as a vulnerable period that requires rest and protection. These cultural beliefs have been reported as the major barriers for increasing physical activity level in Chinese pregnant women^26,27^. However, our study findings showed that physical activity level was generally low among Chinese pregnant women in Hong Kong. More importantly, our findings revealed that giving advice on physical activity alone by the exercise instructor in the LMP did not produce increased physical activity level among the LMP participants. In view of the findings, a more intensive physical activity component, such as the possibility of supervised exercise programs or groups could be considered in the LMP as to pose some more impacts on the measured outcomes. However, the intensity, types and frequency of the exercise program, and the best model of delivery of the exercise program as well as the target group that would benefit most from the exercise program in terms of the local context remain to be identified. Fourth, consistent with the findings from previous studies that higher pre-pregnant BMI was associated with higher risk of GDM incidence and lower total GWG, whether the intervention approach should start before pregnancy warrants further investigation.

Our study has several limitations. First, the recruitment was done in one hospital and the results may not be generalized. Second, there is no routine measurement of infant’s length and head circumference at birth in the study hospital, and some mother-infant pairs had been discharged from the hospital prior to the anthropometric measurement by the research team. Therefore, anthropometric data of some infants were missing. Third, some mothers chose to give birth in the private hospital, thus most birth and infant data of these mothers were self-reported by the mothers based on the available record from the private hospital. Fourth, absence of any difference in most outcomes between the two groups might be due to the inadequate sample size used. Last, the IPAQ used was not specifically designed for pregnant population. Other feasible methods, such as the use of accelerometer for measuring physical activity among pregnant women may be more accurate.

In conclusion, the LMP proposed in the study did not influence GDM risk and other maternal and infant outcomes in Chinese pregnant women at risk of GDM in Hong Kong. Pregnant women with higher adherence to the LMP showed a more desirable dietary composition and energy intake, and a lower proportion of excessive GWG. The potential effect of the LMP on improving maternal outcomes, in particular GWG, may require study with a larger sample size and stronger exercise component. Barriers for Chinese pregnant women in Hong Kong to make lifestyle changes and best strategies to faciliate such changes also warrant further investigations.

## Electronic supplementary material

Supplement 1

## Data Availability

All data generated or analysed in the study are included in this published article.
