# Supplementary material for: Randomized trial examining effectiveness of lifestyle intervention in reducing gestational diabetes in high risk Chinese pregnant women in Hong Kong
Source: Sci Rep. 2018 Sep 14;8:13849. doi: 10.1038/s41598-018-32285-6 (PMC6138708; doi:10.1038/s41598-018-32285-6)
Supplement: Supplementary file 1 — Supplement 1 [file 41598_2018_32285_MOESM1_ESM.doc]

##

**The Chinese University of Hong Kong**

**Clinical Study Protocol**

**A randomized controlled trial examining the effectiveness of a lifestyle intervention in reducing gestational diabetes mellitus in high risk Chinese pregnant women in Hong Kong**

**Principal Investigator: Prof Ruth Chan Suk-Mei**

**Research Assistant Professor**

**Department of Medicine and Therapeutics**

**The Chinese University of Hong Kong**

**1. BACKGROUND**

**Global burden of gestation diabetes mellitus (GDM)**

Gestational diabetes mellitus (GDM) is defined as a type of diabetes first diagnosed during pregnancy (1). With the increasing prevalence of obesity and diabetes worldwide, in particular type 2 diabetes among women, there have been increasing number of women with GDM (2). GDM affects 1% to 14% of pregnancies, depending on the population studied and the diagnostic tests used (3). Maternal glucose level has been associated with a range of adverse maternal and pregnancy outcomes. Women with GDM have an increased risk for developing the metabolic syndrome and early markers of vascular diseases such as disturbed endothelial function (4). GDM has also been linked with higher rates of cesarean sections, induced deliveries, shoulder dystocia and macrosomia, and pathogenesis in the offspring of overweight and metabolic syndrome (4).

Accumulating evidence suggests that an adverse environment during in utero or postnatal periods are related to the development of obesity and associated disorders later in life. Overseas findings of human studies accumulate that intrauterine or postnatal nutrition has a major impact not only on immediate outcomes, such as growth, but also on long-term health later in life (5). Therefore, the importance of timely intervention at early life in reducing the lifetime burden from non-communicable diseases (NCDs) has been promoted (6).

**Nutritional status, GDM prevalence and its associated impacts on the maternal and child health in Hong Kong**

There is a tendency towards over- rather than under-nutrition among pregnant women in Hong Kong. Using body mass index (BMI) cutoff of World Health Organization (WHO) for Asian, 9% pregnant women were underweight (BMI <18 kg/m^2^) whereas 31.7% were overweight or obese (BMI >23 kg/m^2^) in Hong Kong (7). The prevalence of GDM in the early 90s was 14.2% among local pregnant women defined using the 1998 WHO criteria (8). This prevalence is expected to increase alongside with the rising epidemic of obesity and DM in Hong Kong in the past decades. Similar to overseas observations, common risk factors for GDM among local pregnant women include increased maternal age, high pre-pregnancy weight status, high maternal BMI, and excessive gestational weight gain (7, 9, 10). However, limited local data have documented the influence of maternal nutritional status on the short- and long-term health of the mothers and their children.

Until recently, Tam and colleagues have published data on this aspect based on the findings of the GDM follow-up study and the “Hyperglycemia and Adverse Pregnancy Outcome (HAPO)” study. Their findings support overseas observations that in utero nutritional status has a great impact on both the short- and long-term health of the mothers and the offspring. Their data showed that maternal GDM increased the offspring’s cardiometabolic risk, and maternal hyperinsulinemia was an independent predictor of abnormal glucose tolerance in childhood and cardiometabolic risk at early adolescence (11). Preliminary findings of the HAPO study suggested that maternal hyperglycemia at pregnancy may influence the children’s degree of hyperglycemia at age 7 years (12). Regarding the GDM associated health impacts on mothers, recent local data reveal that women with history of GDM have increased risk of future progression to abnormal glucose tolerance, DM and hypertension at 15 years postpartum (13). These findings definitely support the importance of timely intervention in the prenatal period in reducing the lifetime burden from NCDs.

**Lifestyle intervention at early pregnancy as a feasible mean to improve maternal GDM and weigh gain**

Pre-pregnancy weight status is strongly associated with most adverse outcomes of obese pregnancies, therefore timely interventions for weight management and GDM reduction would be ideally planned prior to pregnancy. However, this would be difficult in view of the likelihood of some unplanned pregnancies and the low compliance with lifestyle recommendations among women planning pregnancy (14). The antenatal period is therefore considered an alternative ideal time for interventions as mothers are motivated to make changes to optimize their own health and that of the baby (14). Currently there is no consensus regarding the best intervention for weight management and GDM reduction during pregnancy. The quality of most published studies so far is mainly poor (15). However, available evidence appears to suggest that antenatal lifestyle interventions, in particular dietary interventions is associated with restricted gestational weight gain and a trend towards a reduced prevalence of GDM in overweight or obese pregnant women (16). More importantly, dietary intervention is safe and potentially cost effective and there is no evidence that the lifestyle interventions are associated with adverse maternal or fetal outcomes (15). Therefore, well designed randomized trials with standardized behavioral interventions are warranted.

**Previous work done by us on lifestyle interventions and GDM**

The investigator team has developed a lifestyle modification program (LMP) in Hong Kong. The program is clinically proven and developed based on the Cognitive Behaviour Therapy (CBT) concept, using motivational interviewing and behavioural modification, to accompany improving knowledge and daily practice regarding diet and physical activity. It is a dietitian-led intervention which targets at empowering participants to make lifestyle behavioural changes to achieve long-lasting impact. A varied balanced diet with an emphasis on fruit and vegetables, moderate-carbohydrate, low-fat, low-glycaemic index (GI) and low-calorific products in appropriate portions is encouraged. Participants of the LMP will also be arranged to see an exercise instructor at least once during the program. The exercise instructor will review the participant’s medical history and exercise habits, and design a suitable exercise regime for the participant. Compliance of over 70% has been documented in the LMP among general overweight or obese adults. The program is effective in weight management in Chinese obese adults with and without DM (17), normalizing liver fat in patients with non-alcoholic fatty liver disease (18), and producing a long-lasting impact on lifestyle changes even after the cessation of the program (17). The investigator team has routinely provided LMP for pregnant women of different gestational age. Preliminary findings based on 12 pregnant women commencing LMP between 4 and 12 gestational weeks from January to June 2013 showed that LMP is effective in preventing excessive weight gain without posing any adverse effects on the mothers and the foetus. Ten out of these 12 women showed acceptable weight gain. One of the collaborators (TWH) and colleagues have also published papers covering the risk factors for GDM and the influence of GDM on the short- and long-term health of the mothers and their children. Their findings support overseas observations that maternal hyperglycaemia has a great impact on both the short- and long-term health of the mothers and the offspring (11-13).

Several local aspects have urged the need of this proposed study. Firstly, maternal obesity and GDM are prevalent in Hong Kong. Secondly, our intervention will be planned as early as at or before 12 weeks gestation, which supports the importance of early and timely intervention in reducing GDM and preventing excessive weight gain. Thirdly, to our knowledge, no study has examined the feasibility of a lifestyle intervention in pregnant women in Hong Kong. Based on these considerations, we propose the present study to determine the potential efficacy of a lifestyle intervention in early pregnancy in reducing GDM in high risk Chinese pregnant women in Hong Kong.

**2. AIM**

1. To compare the effectiveness of a lifestyle intervention in early pregnancy with the usual antenatal care in reducing the prevalence of GDM (primary outcome) and the proportion of infant born large for gestational age (LGA) and macrosomia (secondary outcomes) in high risk Chinese pregnant women in Hong Kong
2. To explore the effectiveness of a lifestyle intervention in early pregnancy as compared to the usual antenatal care in improving other obstetric maternal outcomes and fetal and neonatal outcomes (tertiary outcomes) in high risk Chinese pregnant women in Hong Kong

**3. PLAN OF INVESTIGATION**

| **Study design**   | This will be a parallel group, single-blind randomized controlled trial. The study will last for 24 months and will be divided into 5 phases including study preparation (1^st^ to 3^rd^ month), subject recruitment (4^th^ to 9^th^ month), data collection (4^th^ to 21^st^ month), data entry (4^th^ to 22^nd^ month), data analysis and report writing (19^th^ to 24^th^ month). The study will be conducted in accordance with the Declaration of Helsinki. | | --- | |  |   **Subjects**  **Subject recruitment** - Subjects with gestational age <=12 weeks will be recruited using a convenience sampling method at the first antenatal (AN) booking visit of the Prince of Wales Hospital (PWH). Subjects will be eligible for the study if they fulfil the following inclusion and exclusion criteria. The research staff will screen clients attending the AN clinic, check for eligible subjects, explain study details and invite them to join the study. Written consent will be obtained from all subjects.  **Inclusion criteria**   - Chinese origin - Reside normally in Hong Kong - Singleton pregnancy - Gestational age <= 12 weeks at the time of recruitment - Could speak and understand Chinese - Fulfill at least one of the following criteria for high risk of GDM at the time of recruitment based on the update hospital protocol implemented since 1^st^ May 2014   - Maternal age >= 35 years old at expected date of confinement.   - Prior history of GDM or birth of child >= 4 kg   - Pre-pregnant BMI or BMI at 1^st^ trimester >= 25 kg/m^2^   - Family history of diabetes at 1^st^ degree relatives - Willing to give informed written consent and follow the study procedures   **Exclusion criteria**   - Concurrent participation in any clinical trial or study - With renal, liver or thyroid dysfunction, cognitive impairment, or any other indication of a major medical or psychological illness, as judged by the investigators as ineligible to participate the study - Multiple pregnancies - Pre-existing DM - With physical restriction that prevents from exercising - Substance abuse |
| --- | --- | --- |
|  |
| **Methods**  **Study outcomes –** The primary outcome will be the proportion of subjects developed GDM at 24-28 weeks gestation in in both groups as measured using a 75 g 2-h oral glucose tolerance test (OGTT). The secondary outcomes will include the proportion of neonates born with LGA (>=95^th^ percentile of the customized birth weight) and macrosomia (>=4 kg at birth) in both groups. The secondary outcomes are chosen since infants born with larger size, in particular those born with GDM mothers were associated with higher future metabolic risk as compared to those born appropriate-for-gestational age with non-GDM or GDM mothers (19).  **Sample size calculation** – Sample size was calculated based on the primary outcome, i.e. the proportion of subjects developed GDM at 24-28 weeks of gestation between the two study groups. A previous lifestyle intervention trial in obese pregnant women showed that 6% subjects developed GDM in the intervention group compared to 29% in control group (p<0.05) (20). Based on these figures, the sample size estimates for a two-sided test will be 42 per group with power of 80% at a 5% significance level to detect an 83% reduction in the odds of decreased GDM in the intervention group when compared to the control group (20). However, a more conservative sample size estimate is used as the proposed lifestyle modification program (LMP) has been modified with less frequent dietetic sessions compared with that used in our previous publications. Therefore, a sample size of 73 per group with power of 90% at a 1% significance and two-sided test will be used. Assuming 30% lost to follow-up rate and 5% miscarriage rate, a final sample size of 110 subjects per group will be required.  **Trial procedures** – Eligible subjects will be randomized in 1:1 ratio to participate in the LMP or receive usual care from the time of recruitment (i.e. <= 12 weeks gestaion) till 24 weeks gestation. Randomization will be performed through the use of a computer-generated list of random numbers in blocks of 6 by a study coordinator. Treatment assignments will be concealed in consecutively-numbered sealed envelopes, which will be opened sequentially upon subject enrollment. The interventionists (the dietitian and the exercise instructor), the subjects and the study coordinator will not be blinded to the treatment assignment. However, the interventionists will not take outcome measurements. Subjects will be reminded not to reveal information about their intervention to the outcome assessors, clinicians and nurses of routine antenatal and postnatal care. All investigators, outcome assessors, clinicians and nurses of routine antenatal and postnatal care will be blinded to the treatment assignment.  Data will be collected by trained research staff at six time points, namely <=12 weeks gestation (baseline/T0), 16-20 weeks gestation (T1), 24-28 weeks gestation (T2), 35-37 weeks gestation (T3), within 48 hours of delivery (T4), and 6-8 weeks postpartum (T5). These time points are scheduled mostly together with the routine AN care visits to minimize subjects’ burden for extra visits for the study. A cash of HK$50 per visit will be payable to each subject at T1, T2, T3 and T5.  **Intervention group** – In addition to receive routine antenatal care, subjects randomized to the intervention group will participate in a dietitian-led LMP from the first AN booking (i.e. <=12 weeks gestation) to 24 weeks gestation. The original LMP is based on a strategy of increasing energy expenditure and reducing caloric intake using lifestyle behavioral change to achieve long-lasting impact. It generally requires participants to attend dietary consultation sessions weekly in the first 3-4 months, and monthly in the subsequent months, and to see an exercise instructor at least once during the program. However, considering that frequent visits may be a burden for pregnant women but frequent contacts in early stage of pregnancy are important for effective lifestyle intervention (21); the original LMP will be modified in several aspects. Firstly, consultations will be scheduled bi-weekly instead of weekly in the first 2 months of the program and monthly to the end of the program (i.e. 24 weeks gestation). Secondly, some original face-to-face consultations in the first two months will be replaced by telephone consultations since the effectiveness of high-frequency (i.e. weekly) telephone contact with a dietitian was comparable to high-frequency face-to-face lifestyle counseling in supporting obese patients trying to lose weight (21). Thirdly, the consultation sessions will be mostly scheduled on the same day of the routine AN visits of the subjects.  At the first session (about 1 hour), the dietitian will carry out a complete behavioral assessment, covering important areas such as the subject’s pre-pregnant and current weight status, eating and lifestyle patterns, specific eating-related behaviors, traditional food taboos in pregnancy, knowledge of risks associated with current eating patterns, and concerns and feelings about specific lifestyle changes. The dietitian will review the subject’s medical, pregnant and birth history, and the weight gain in previous pregnancy. Moreover, the dietitian will review the fetal growth and the subject’s weight gain of current pregnancy, and discuss the specific dietary and lifestyle advices to achieve a desirable weight status with the subjects.  In the follow-up consultation sessions (about 20 minutes), the dietitian will review the subject’s dietary and lifestyle practices and provide recommendations. Each subject will be given an individualized menu plan aiming at achieving a varied balanced diet and a desirable fetal growth and maternal weight throughout pregnancy. The dietary component of the menu plan will be based on the American Dietetic Association’s recommendations (22). A varied balanced diet with an emphasis on fruit and vegetables, and moderate-carbohydrate, low-fat, low-GI and low-calorific products in appropriate portions will be encouraged. Each subject will be provided with two booklets, one for food portion size exchange and tips for eating out, and another listing the low-GI food options and meal plans (GI <55). Moreover, advice on use of dietary supplements and managing food cravings, nausea, vomiting and constipation will be given. Techniques for coping at-risk situations such as parties and festival celebrations will be taught. Recipes will be provided to the subjects to encourage healthy cooking. The dietitian will also encourage the subjects to send their enquiries to the dietitian by emails.  Besides, subjects will be encouraged to see an exercise instructor at least once during the LMP, which will be tried to schedule on the same day as the dietitian appointment and/or AN visits for subjects’ convenience. The exercise instructor will arrange individualized follow-up session based on his/her clinical judgment. Subjects will be encouraged to request for exercise consultations as often as they feel necessary. Ongoing support from the exercise instructor will also be available by phone calls or emails. During the exercise consultation (about 30 minutes), the exercise instructor will review subject’s medical history as well as pre-pregnant and current exercise habits, and assess subject’s fitness level and musculoskeletal problems. The exercise instructor will design a suitable exercise regime for the subject. Medical clearance is recommended for women who have been sedentary before pregnancy or who have a medical condition. The exercise instructor will use the PARmed-X for Pregnancy as a guideline for health screening for the subjects prior to participation in a prenatal fitness class or other exercise, and use the information as a reference to decide the individualized exercise prescription and the program safety. During the first trimester, pregnant women who were previously exercising may continue but modify the exercise regime in accordance with general physiological changes, like high to low impact exercise, while inactive women should refrain from exercise until the second trimester. After the first trimester, subjects will generally be advised to do a 30-minute of easy to moderate intensity of low impact aerobic exercise, including brisk walking, stationary bike, swimming or walking in water for at least three times a week. Subjects will be asked to work out at a manageable intensity which talking could be done without difficulty and with slightly elevated heart rate and breathing rate. For easy indication, the exercise instructor will ask the subjects to weigh their rate of exertion by using the 10-point Borg scale and level 1-4 indicates the exercise intensity as easy to somewhat hard. The exercise instructor will demonstrate techniques for static stretching (avoiding muscle pain) of most muscle groups (upper and lower limbs, neck and trunk muscles) to the subjects. Moreover, the exercise instructor will show to the subjects how to do resistance training, covering the major muscle groups of the arms, abdomen and legs to promote good posture, prevent low back pain and lower limb join problems. Subjects will receive written exercise prescription with frequency and duration targets, and brochures including examples for adequate exercise. Information regarding precautions of participation in a prenatal fitness class or other exercise, such as supine position over 16 weeks, overheating, breath holding and long standing will be provided to the subjects. Information regarding warning signs, such as excessive shortness of breath, dizziness and fainting to terminate exercise and when medical advice should be sought will also be provided.  To check the adherence to both diet and exercise intervention, 3-day dietary records, daily activity log sheets, pedometer readings and attendance data will be used. Participants’ adherence to both the diet and exercise intervention will be assessed by a check-list and an adherence score to each of the diet component and the exercise component will be generated. 1 score will be given for meeting each of the key recommendations given by the dietitian or the exercise instructor. Higher score indicates the participant being more adherent to the LMP.  For the diet adherence score, 1 score will be given for meeting each of the criteria below: (i) total energy not exceeding 10% of the diet plan; (ii) % energy from fat 20-30%; (iii) % energy from protein within the range of 15-20%; (iv) % energy from carbohydrate 50-60%; (v) consumption of fruit > 160g; (vi) consumption of vegetables > 240g (equivalent to 1-2 servings of fruits and 3-4 servings of vegetables); (vii) regular meal consumption as prescribed by dietitian and (viii) “Avoid food (e.g. high fat or high sugar foods/glycaemic index foods)” not being consumed. The scores will be summed up to give a dietary adherence score, ranging from 0 to 8 and a maximum of 24 scores (8 scores x 3 day) for each follow-up time point. Adherence to dietary intervention will also be assessed by calculating the percentage attendance to the intervention sessions.  Regarding the physical activity adherence, the score will be assessed based on the IPAQ-C data. Two scores will be given if 80% of the recommended volume of exercise (frequency x duration) was met during the week of follow up (7 days) while one score will be given if 50% of the recommended volume of exercise was achieved. Zero score will be given for those who did not perform any easy to moderate intensity of low impact aerobic exercise. Number of exercise consultation sessions attended by the subjects will also be documented.  To measure physical activity level more objectively, we will also use pedometers as an objective measurement to tally steps taken by the participants. However, due to limited funding budget, only a sub-group (25%) of subjects in each arm, who will be randomly chosen and will be given a pedometer to record daily step counts for up to seven consecutive days at various time points. The pedometer readings will be filled daily for 7 days at each time point.  **Control group** - Subjects in the control group will receive routine antenatal care shared between the Li Ka Shing outpatient clinic of the PWH and the Maternal and Child Health centres. The subjects will be provided with an educational pamphlet on diet and exercise during pregnancy and offered optional antenatal classes which subjected to quotas availability. An updated protocol has been implemented in the PWH since 1st May 2014 to standardize the screening, diagnosis and management of GDM.  **Measurements –** Various maternal and fetal/neonatal data will be collected using standardized methods and questionnaires, and retrieved from the hospital record.   - **Maternal physical examination measurements:** Weight, height, blood pressure and heart rate will be measured using standardized methods, and pre-pregnancy weight will be self-reported. The total gestational weight gain will be calculated as the difference in the weight measured on the day of delivery and self-reported pre-pregnancy weight. - **Maternal Laboratory assessments:** A 75 g 2-h OGTT will be done at 24-28 weeks gestation on routine AN care basis. GDM will be diagnosed using the modified WHO criteria. In the study hospital, a new protocol of diagnosis of GDM has been implemented since 1^st^ May 2014. Only the fasting plasma glucose (FPG) and 2-h plasma glucose (PG) results of the OGTT instead of the fasting, 1-h and 2-h PG results will be used as diagnostic criteria for GDM due to resource constraints. Therefore, the primary outcome (i.e. GDM) will be diagnosed between 24-28 weeks of gestation if one or more of the following criteria are met: (i) FPG 5.1-6.9 mmol/L; (ii) 2-hour PG 8.5-11.0 mmol/L following a 75g oral glucose load. Extra OGTT will be repeated at 6-8 weeks postpartum for those who have abnormal OGTT results at 24-28 weeks gestation. - **Demographic and other lifestyle habits**, such as mother’s age, occupation, family income, smoking status and alcohol use will be collected using a standardized questionnaire at baseline. Smoking status and alcohol use will be asked again at 35-37 week gestation to check for any changes in these habits during pregnancy. - **Medical history, past/current obstetric history, medication use** will be retrieved from the hospital record and collected using a questionnaire at baseline and at 35-37 weeks gestation. - **Dietary intakes, physical activity level and use of supplements** will be assessed. A 3-day diet record including one weekend day and two weekdays will be used to assess subject’s diet. Daily nutrient intake and consumption of food group of all subjects at various time points will be calculated using the nutrition analysis software Food Processor Nutrition analysis and Fitness software version 8.0 (ESHA Research, Salem, USA) including local foods selected from food composition tables from China and Hong Kong. Since there has been no validated Chinese questionnaire for measuring physical activity level for pregnant women, the present study will use a Chinese version of the International Physical Activity Questionnaire (IPAQ-C) which has been validated among general Chinese population (23). Moreover, a general physical activity questionnaire capturing special physical activity patterns of pregnant women will be asked. A sub-group (25%, n=28) of subjects in each arm, who will be randomly chosen and be be given a pedometer (Yamax brand) to record daily step counts for up to seven consecutive days at various time points. The pedometer readings will be filled daily for 7 days at each time point. Data on use of supplements will be collected by asking subjects to specify the brand, the frequency, and the dosage of supplements used. - **Perceived support from the dietitian and the exercise instructor** will be assessed at 24-28 weeks of gestation (T2) using the Health Care Climate Questionnaire (HCCQ), a 15-item patient-rated measures on a scale of 1 (strongly disagree) to 7 (strongly agree) related to their perceived supportiveness of health care providers. HCCQ is validated among various settings. The Chinese version was validated with high internal reliability (0.93) (24). - **Perinatal and obstetric outcomes, neonatal outcomes and complications** will be retrieved from the hospital record. Perinatal and obstetric outcomes and complications will include GDM as measured by OGTT at 24-28 weeks gestation, preeclampsia, gestational hypertension, Caesarean section and preterm delivery, and gestation at delivery (week). Fetal and neonatal outcomes will include fetal ultrasound, Apgar score at 1 minute and 5 minutes, neonatal weight and length, head circumference, LGA, macrosomia and shoulder dystocia. |
|  |
| **Data processing and analysis**  Data will be presented as mean (standard deviation), median (inter-quartile range) for skewed variables and frequency (percentage) as appropriate. Skewed variables will be corrected by necessary transformations before statistical analyses. Between-groups comparisons at baseline and after intervention will be made using the Student’s t test, Chi-square test or Fisher’s exact test, as appropriate. Multilevel models will be used to compare differences between the 2 groups for all measured outcomes including GDM prevalence, proportion of infants born LGA and macrosomia, anthropometric measures and other obstetric maternal outcomes and fetal and neonatal outcomes at baseline and 24-28 weeks gestation or at delivery with adjustment for age and other covariates. Multilevel model can be used to analyse both longitudinal continuous and discrete data (25). All statistical tests are two-sided and a p-value <0.05 is considered statistically significant. SPSS for Windows software (version 24.0, SPSS Inc., Chicago, IL, USA) will be used for the statistics. |

**4. Subject withdrawal**

A subject must be withdrawn from the study if she withdraws consent. Subjects who (1) experience adverse events, or (2) have pre-existing violation of entry criteria may remain in the study unless the investigator determines that it is not in the subject’s best interest to continue. The specific reason for withdrawal should be indicated.

Subjects who withdraw from the study are invited to attend the follow-up measurement at 24-28 week gestation to determine the primary outcome. If a subject does not return for a scheduled visit, every effort should be made to contact the subject. In any circumstance, every effort should be made to document subject outcome, if possible.

**5. References**

1. Metzger BE. Summary and recommendations of the Third International Workshop-Conference on Gestational Diabetes Mellitus. Diabetes. 1991;40 Suppl 2:197-201.

2. Roglic G. Diabetes in women: the global perspective. International Journal of Gynaecology & Obstetrics. 2009;104 Suppl 1:S11-3.

3. Hunt KJ, Schuller KL. The increasing prevalence of diabetes in pregnancy. Obstetrics & Gynecology Clinics of North America. 2007;34(2):173-99.

4. Kaaja R, Rönnemaa T. Gestational Diabetes: Pathogenesis and Consequences to Mother and Offspring. The Review of Diabetic Studies. 2008;5(4):194-202.

5. Tarry-Adkins JL, Ozanne SE. Mechanisms of early life programming: current knowledge and future directions. American Journal of Clinical Nutrition. 2011;94(6 Suppl):1765S-71S.

6. Symonds ME, Mendez MA, Meltzer HM, Koletzko B, Godfrey K, Forsyth S, et al. Early life nutritional programming of obesity: mother-child cohort studies. Annals of Nutrition & Metabolism. 2013;62(2):137-45.

7. Leung TY, Leung TN, Sahota DS, Chan OK, Chan LW, Fung TY, et al. Trends in maternal obesity and associated risks of adverse pregnancy outcomes in a population of Chinese women. BJOG: An International Journal of Obstetrics & Gynaecology. 2008;115(12):1529-37.

8. Ko GT, Tam WH, Chan JC, Rogers M. Prevalence of gestational diabetes mellitus in Hong Kong based on the 1998 WHO criteria. Diabetic Medicine. 2002;19(1):80.

9. Lao TT, Tam KF. Gestational diabetes diagnosed in third trimester pregnancy and pregnancy outcome. Acta Obstetricia et Gynecologica Scandinavica. 2001;80(11):1003-8.

10. Lao TT, Ho LF, Chan BC, Leung WC. Maternal age and prevalence of gestational diabetes mellitus. Diabetes Care. 2006;29(4):948-9.

11. Tam WH, Ma RC, Yang X, Li AM, Ko GT, Kong AP, et al. Glucose intolerance and cardiometabolic risk in adolescents exposed to maternal gestational diabetes: a 15-year follow-up study. Diabetes Care. 2010;33(6):1382-4.

12. Tam WH, Ma RC, Yang XL, Chan JC, Lao TT, Yip GW, et al. The correlation between antenatal OGTT glycemic levels and of children's glycemic levels at OGTT at 7 yeas of age. The 6th International Symposium on Diabetes & Pregnancy; 2011 3/24/2011; Kenes International, Salzburg, Austria. 2011.

13. Tam WH, Ma RC, Yang X, Ko GT, Lao TT, Chan MH, et al. Cardiometabolic risk in Chinese women with prior gestational diabetes: a 15-year follow-up study. Gynecologic & Obstetric Investigation. 2012;73(2):168-76.

14. Inskip HM, Crozier SR, Godfrey KM, Borland SE, Cooper C, Robinson SM, et al. Women's compliance with nutrition and lifestyle recommendations before pregnancy: general population cohort study. BMJ. 2009;338:b481.

15. Thangaratinam S, Rogozinska E, Jolly K, Glinkowski S, Roseboom T, Tomlinson JW, et al. Effects of interventions in pregnancy on maternal weight and obstetric outcomes: meta-analysis of randomised evidence. BMJ. 2012;344:e2088.

16. Oteng-Ntim E, Varma R, Croker H, Poston L, Doyle P. Lifestyle interventions for overweight and obese pregnant women to improve pregnancy outcome: systematic review and meta-analysis. BMC Medicine. 2012;10:47.

17. Woo J, Sea MM, Tong P, Ko GT, Lee Z, Chan J, et al. Effectiveness of a lifestyle modification programme in weight maintenance in obese subjects after cessation of treatment with Orlistat. Journal of Evaluation in Clinical Practice. 2007;13(6):853-9.

18. Wong VWS, Chan RSM, Wong GLH, Cheung BHK, Chu WCW, Yeung DKW, et al. Community-based lifestyle modification programme for non-alcoholic fatty liver disease: A randomized controlled trial. . Journal of Hepatology. 2013;59:536-42.

19. Boney CM, Verma A, Tucker R, Vohr BR. Metabolic syndrome in childhood: association with birth weight, maternal obesity, and gestational diabetes mellitus. Pediatrics. 2005;115(3):e290-6.

20. Quinlivan JA, Lam LT, Fisher J. A randomised trial of a four-step multidisciplinary approach to the antenatal care of obese pregnant women. Australian & New Zealand Journal of Obstetrics & Gynaecology. 2011;51(2):141-6.

21. Digenio AG, Mancuso JP, Gerber RA, Dvorak RV. Comparison of methods for delivering a lifestyle modification program for obese patients: a randomized trial. Annals of Internal Medicine. 2009;150(4):255-62.

22. Wheeler ML, Franz M, Barrier P, Holler H, Cronmiller N, Delahanty LM. Macronutrient and energy database for the 1995 Exchange Lists for Meal Planning: a rationale for clinical practice decisions. J Am Diet Assoc. 1996 Nov;96(11):1167-71.

23. Macfarlane DJ, Lee CC, Ho EY, Chan KL, Chan DT. Reliability and validity of the Chinese version of IPAQ (short, last 7 days). Journal of Science & Medicine in Sport. 2007;10(1):45-51.

24. Chan DKC, Hagger MS, Spray CM. Treatment motivation for rehabilitation after a sport injury: Application of the trans-contextual model. Psychology of Sport and Exercise. 2011;12(2):83-92.

25. Singer J, Willett J. Applied longitudinal data analysis: modeling change and event occurrence. New York: Oxford University Press; 2003.
